# Improved Face Image Super-Resolution Model Based on Generative Adversarial Network

**DOI:** 10.3390/jimaging11050163

**Published:** 2025-05-19

**Authors:** Qingyu Liu, Yeguo Sun, Lei Chen, Lei Liu

**Affiliations:** 1 School of Computer Science, Huainan Normal University, Huainan 232038, China; leichen@hnnu.edu.cn (L.C.); liulei@hnnu.edu.cn (L.L.); 2 School of Finance and Mathematics, Huainan Normal University, Huainan 232038, China; yeguosun@126.com

**Keywords:** super-resolution, generative adversarial network, attention mechanism, residual module

## Abstract

Image super-resolution (SR) models based on the generative adversarial network (GAN) face challenges such as unnatural facial detail restoration and local blurring. This paper proposes an improved GAN-based model to address these issues. First, a Multi-scale Hybrid Attention Residual Block (MHARB) is designed, which dynamically enhances feature representation in critical face regions through dual-branch convolution and channel-spatial attention. Second, an Edge-guided Enhancement Block (EEB) is introduced, generating adaptive detail residuals by combining edge masks and channel attention to accurately recover high-frequency textures. Furthermore, a multi-scale discriminator with a weighted sub-discriminator loss is developed to balance global structural and local detail generation quality. Additionally, a phase-wise training strategy with dynamic adjustment of learning rate (Lr) and loss function weights is implemented to improve the realism of super-resolved face images. Experiments on the CelebA-HQ dataset demonstrate that the proposed model achieves a PSNR of 23.35 dB, a SSIM of 0.7424, and a LPIPS of 24.86, outperforming classical models and delivering superior visual quality in high-frequency regions. Notably, this model also surpasses the SwinIR model (PSNR: 23.28 dB → 23.35 dB, SSIM: 0.7340 → 0.7424, and LPIPS: 30.48 → 24.86), validating the effectiveness of the improved model and the training strategy in preserving facial details.

## 1. Introduction

Face super-resolution (FSR) focuses on synthesizing high-resolution (HR) facial textures from low-resolution (LR) inputs. This task has gained considerable attention in computer vision research to address real-world challenges [1,2,3]. This field holds significant application value in security surveillance [4], medical image enhancement [5], and remote sensing image analysis [6]. However, due to the unique anatomical structure of human faces and the high sensitivity of the human visual system to facial details, FSR presents greater technical challenges compared to general image super-resolution. These challenges primarily include the precise restoration of high-frequency texture details and the maintenance of natural facial structure coherence. The inherent loss of high-frequency information in LR images further impedes conventional methods from generating outputs that meet human visual perception standards for high-quality reconstruction.

Early image SR methods primarily relied on interpolation algorithms [7,8,9] or reconstruction-based approaches [10,11], but these techniques often produced local blurring and artifacts when enlarging images at high magnification factors. The advent of the convolutional neural network (CNN) [12] significantly advanced SR technology. Dong et al. [13] pioneered the development of SRCNN, establishing an end-to-end model for SR. Their approach leverages CNN to transform LR inputs into HR outputs. However, it struggled to capture intricate image details. To accelerate reconstruction speed, FSRCNN employed deconvolution techniques and parameter-sharing strategies [14]. In 2016, following the introduction of the ResNet [15], Kim et al. [16] proposed the Very Deep Super-Resolution (VDSR) model. This approach enhances feature extraction by increasing network depth and accelerated training via residual learning. However, it demands substantial computational resources and exhibits limitations in recovering intricate textures. Lim et al. [17] introduced the EDSR model, leveraging deep residual networks for SR, but its large parameter count and high computational costs limited practical deployment. Subsequently, RCAN [18] incorporated the channel attention mechanism [19] to enhance feature representation, achieving breakthrough performance in PSNR. Nevertheless, methods optimized via mean squared error (MSE) generally exhibit over-smoothing artifacts in generated images.

To enhance image SR quality, Ledig et al. pioneered the integration of generative adversarial network (GAN) [20] into SR tasks by proposing the SRGAN model [21]. ESRGAN [22] further optimized network architecture through Residual-in-Residual Dense Blocks (RRDB) and a relativistic average discriminator, significantly improving detail recovery capabilities. Park et al. proposed the SRFeat [23] model, which incorporates two discriminators to mitigate high-frequency noise during the super-resolution process. The RankSRGAN [24] model proposed by Zhang et al. performs super-resolution reconstruction by leveraging image contrast information. In 2020, Shang et al. introduced the RFB-ESRGAN model [25], which incorporates a receptive field block to enhance feature extraction capabilities. The SPSR model [26] proposed by Ma et al. integrates a gradient-based branch structure into the GAN framework to alleviate geometric distortions in image reconstruction. Wang et al. extended this framework by incorporating RRDBs and skip connections to develop Real-ESRGAN [27], enabling end-to-end image inpainting and achieving superior SR performance. Nevertheless, GAN-based methods still face challenges such as training instability, geometric distortions (e.g., facial misalignment), and high-frequency detail degradation. Furthermore, existing approaches typically assume known degradation processes (e.g., Bicubic), whereas real-world LR images exhibit complex degradation patterns, leading to diminished generalization performance. Some studies have attempted to incorporate facial priors for localized detail enhancement [28]. However, errors in prior estimation directly propagate into the SR reconstruction process, compromising model robustness.

To overcome these limitations, we introduce an enhanced GAN for FSR incorporating Multi-scale Hybrid Attention Residual Blocks (MHARB) and an Edge-guided Enhancement Block (EEB). Key contributions are as follows:

(1) We propose a multi-scale hybrid residual block with attention mechanisms (MHARB), which incorporates dual-branch convolution and channel-spatial attention (CBAM). CBAM dynamically adjusts feature weights through channel-wise and spatial attention, thereby improving the feature extraction capability.

(2) To address high-frequency blurring in critical regions (e.g., eyes and teeth), we design an Edge-guided Enhancement Block (EEB). This module first generates a spatial weight map via an edge detection convolution layer (activating edge regions) and then employs channel attention and a Tanh activation function to derive an adaptive detail fusion strategy. This approach achieves localized enhancement of high-frequency regions while mitigating gradient explosion risks.

(3) We propose an improved multi-scale discriminator network architecture. This model progressively processes inputs at three distinct scales and employs a weighted sub-discriminator loss to enforce the generator to simultaneously preserve global consistency and local texture authenticity.

(4) The training process adopts a phased, fine-grained training mechanism. The generator’s Lr is dynamically adjusted using a decreasing strategy (initially large, then small). Simultaneously, the weights of the L1 loss and multi-scale adversarial loss functions are decayed to balance the generation quality of face images with model stability.

## 2. Related Work

### 2.1. GAN

GAN, proposed by Goodfellow in the year of 2014, revolutionized generative modeling by fitting real data distributions through adversarial training. This groundbreaking framework introduced a novel paradigm of “generator-discriminator” competition, thereby redefining the design principles of traditional generative models.

The original GAN has only two sub-networks: a generator (G) and a discriminator (D). The generator’s input is random noise $z\sim P_{z}(z)$ and produces synthesized images G(z), aiming to learn a mapping relationship that approximates a target data distribution $P_{data}(x)$. Conversely, the discriminator receives either real images $x\sim P_{data}(x)$ or generated images G(z) as input and outputs a discriminative probability. It aims to optimize the discriminator’s ability to accurately discriminate between the real and generated images through adversarial training. Mathematically, GAN is formulated as follows:

(1) $${min}_{G}{max}_{D}V\left(D,G\right)=E_{x\sim P_{data}(x)}\left|logD(x)\right|+E_{z\sim P_{z}(z)}\left|\log(1-D(G(z)))\right|$$

During model training, the generator is initially fixed while the discriminator parameters $\theta_{D}$ are updated to maximize $V\left(D,G\right)$. Subsequently, the discriminator is held constant as the generator parameters $\theta_{G}$ are optimized to minimize $V\left(D,G\right)$. This adversarial process is repeated iteratively until the generator converges to or approximates the real images’ distribution.

### 2.2. Attention Mechanisms

#### 2.2.1. Spatial Attention

Spatial attention [29] dynamically adjusts the weights of spatial location information in feature maps to enhance the focus on critical regions. Assuming the input feature map $F\in R^{H\times W\times C}$, the spatial attention weight matrix $M_{s}\in R^{H\times W}$ is formulated as follows:

(2) $$M_{s}=Sigmoid(Conv(7\times 7)(AvgPool\left(F\right);MaxPool(F)))$$

where $AvgPool\left(F\right)$ and MaxPool(F) denote Average-Pooling and Max-Pooling, respectively, while Conv(7×7) represents a convolution layer with a 7×7 kernel size. The derived feature map is represented as:

(3) $$F^{\prime }=F\cdot M_{s}$$

where · denotes element-wise multiplication. During the SR reconstruction of face images, spatial attention enhances feature extraction capabilities in critical regions (e.g., eyes and mouth) while suppressing background noise.

#### 2.2.2. Channel Attention

Channel attention models inter-channel relationships to adaptively adjust the weights of individual channels. Given an input feature map $F\in R^{H\times W\times C}$, the mathematical formulation of the channel attention weight vector $M_{c}\in R^{1\times 1\times C}$ is expressed as follows:

(4) $$M_{c}=Sigmoid(W_{2}\times ReLU(W_{1}\;\times \;AvgPool\left(F\right)))$$

where $W_{1}\in R^{\frac{C}{16}\times C}$ and $W_{2}\in R^{C\times \frac{C}{16}}$ denote the parameters of the Fully Connected Layers. And the output is expressed as:

(5) $$F^{\prime }=F\cdot M_{c}$$

During face image reconstruction, channel attention enhances the response to high-frequency detail channels (e.g., eyes and teeth) by emphasizing critical regions.

#### 2.2.3. Hybrid Attention Mechanism

The hybrid attention mechanism typically combines spatial and channel attention architectures in either serial or parallel configurations to jointly model spatial localization and channel-wise feature importance. A representative implementation, CBAM [30], processes input feature maps through a two-stage pipeline: initially applying channel attention to emphasize critical channels, followed by spatial attention to refine localized features, ultimately yielding refined feature representations.

## 3. Proposed Method

### 3.1. Network Architecture

Our proposed SR GAN comprises a generator and a multi-scale discriminator. To address the challenges of unnatural facial detail restoration and local blurring in existing GAN-based face super-resolution (FSR) methods, our model introduces three key architectural innovations:

(1) The multi-scale hybrid attention residual block (MHARB) can dynamically enhance key facial regions (such as eyes and mouth) while suppressing irrelevant background noise.

(2) The edge-guided enhancement block (EEB) can adaptively generate high-frequency detail, effectively restoring sharp textures in contour areas (such as teeth, hair) without introducing artifacts.

(3) The multi-scale discriminator with weighted loss forces the generator to balance the consistency of the global structure and the authenticity of local details through the weighted sub-discriminator loss.

#### 3.1.1. Generator Design

**Figure 1:**
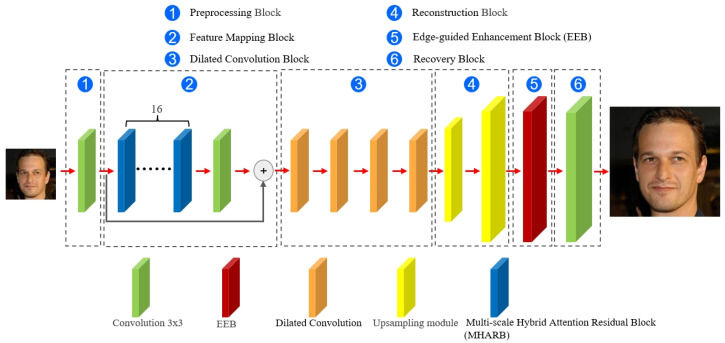
Architectural of the generator.

The generator employs a cascaded architecture to progressively recover high-frequency details through Preprocessing Block, Feature Mapping Block, Dilated Convolution Block, Reconstruction Block, Edge-guided Enhancement Block (EEB), and Recovery Block, as illustrated in Figure 1.

(1) Preprocessing Block

The LR face image is first passed through a 3 × 3 convolutional layer. A LeakReLU activation function is then applied to extract initial shallow features.

(2) Feature Mapping Block

**Figure 2:**
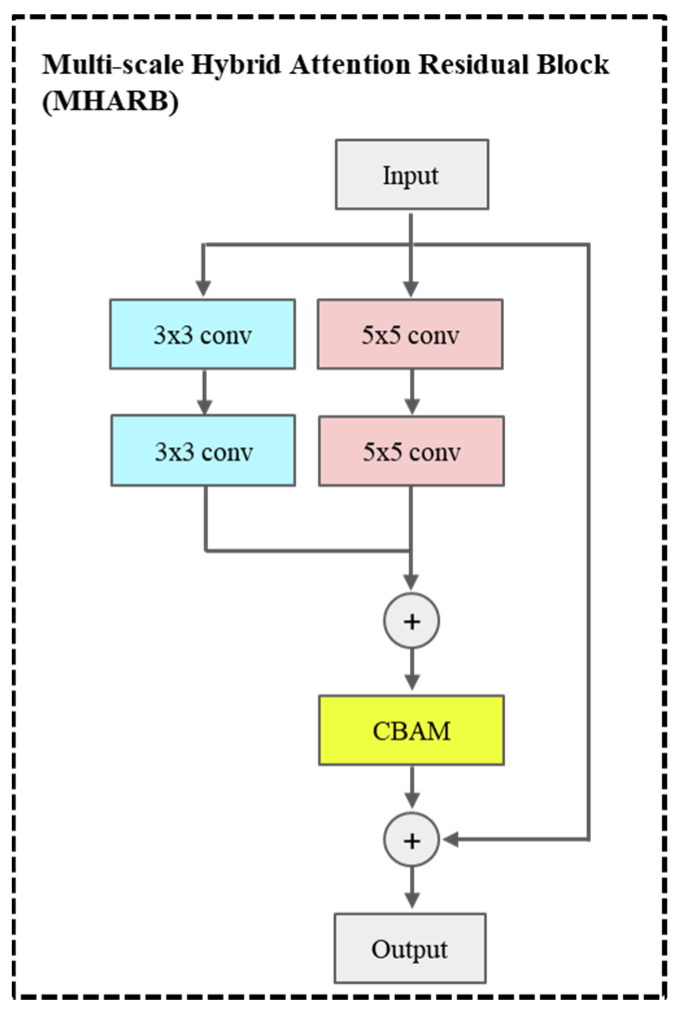
Improved residual block.

Feature Mapping Block uses 16 MHARBs and adds a residual link. The improved residual block, namely, MHARB, incorporates two parallel convolutional pathways with kernel sizes of 3 × 3 and 5 × 5, as illustrated in Figure 2. The outputs of the dual pathways are combined through element-wise summation and subsequently calibrated by the hybrid attention module CBAM. CBAM sequentially performs channel attention and spatial attention to generate a spatial-channel joint weight map, enhancing responses in critical regions. Assuming the input image is denoted as $Input\in R^{C\times H\times W}$, the mathematical formulation is expressed as follows:

(6) $$Y_{1}={Conv}_{3\times 3}^{\left(2\right)}(LeakReLU(InstanceNorm({Conv}_{3\times 3}^{\left(1\right)}(Input))))$$

(7) $$Y_{2}={Conv}_{5\times 5}^{\left(2\right)}(LeakReLU(InstanceNorm({Conv}_{5\times 5}^{\left(1\right)}(Input))))$$

where ${Conv}_{k\times k}^{\left(i\right)}$ denotes the k×k convolution operation at the i-th layer. The convolution kernel in Formula (6) is 3 × 3, and both padding and stride are 1. While the convolution kernel in Formula (7) is 5 × 5 in size, its padding is 2, and the stride size remains 1. Their number of output channels is 64. For Equations (8) and (9), no convolution operation is performed. Therefore, the number of channels in the feature map is 64 for both.

(8) $$Y=CBAM(Y_{1}+Y_{2})$$

The residual block’s output is derived as:

(9) Output=Y+Input

The generator incorporates 16 stacked residual blocks to progressively deepen feature representations.

(3) Dilated Convolution Block

The architecture cascades four groups of dilated convolutional layers with progressively expanding dilation rates ($d_{1}=2,\;d_{2}=4,\;d_{3}=8\;and\;d_{4}=16$). This design captures long-range dependencies in face images through incrementally enlarged receptive fields, thereby mitigating detail loss.

(4) Reconstruction Block

This block employs two groups of upsampling units to upscale the feature map resolution to 256 × 256.

(5) Edge-guided Enhancement Block (EEB)

**Figure 3:**
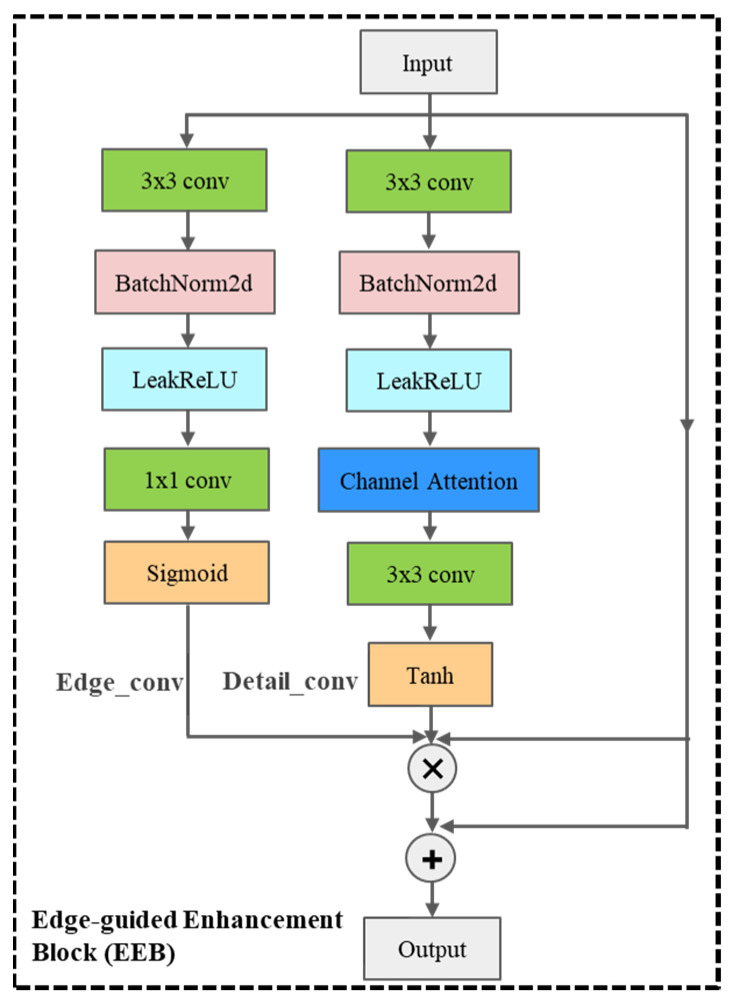
Edge-guided Enhancement Block.

The EEB incorporates an edge detection branch and a detail enhancement branch, structured as two parallel pathways, as illustrated in Figure 3.

EEB consists of two parallel branches: the edge detection branch and the detail enhancement branch, aiming to precisely restore high-frequency texture details through an adaptive fusion strategy. The edge detection branch generates the masks of the edge regions (such as facial contours, eyes, and teeth). The detail enhancement branch can control the enhancement amplitude of high-frequency details.

Edge Detection Branch: A 1 × 1 convolution compresses the input feature map to a single channel, which is then passed by a Sigmoid function to generate an edge mask. Here, high-frequency regions correspond to contours.

Detail Enhancement Branch: A 3 × 3 convolutional layer and channel attention module collaboratively generate channel-wise weights, which are subsequently activated via the Tanh function to output enhancement coefficients for high-frequency regions, emphasizing fine structural details.

The edge mask and detail mask are combined to enhance high-frequency details in edge regions, with the formulation expressed as:

(10) $$Output=Input+Input\cdot M_{edge}\cdot M_{detail}$$

where $M_{edge}\in [0,1]$ and $M_{detail}\in [-1,1]$ denote the edge mask and detail enhancement coefficient, respectively. The former controls the spatial extent of enhancement (i.e., where to amplify), while the latter regulates the intensity of amplification (i.e., how much to amplify). The symbol · denotes element-wise multiplication between tensors.

(6) Recovery Block

This block has only one convolutional layer. The feature map is reconstructed as a 3-channel HR image after a 3 × 3 convolutional layer.

#### 3.1.2. Discriminator Design

**Figure 4:**
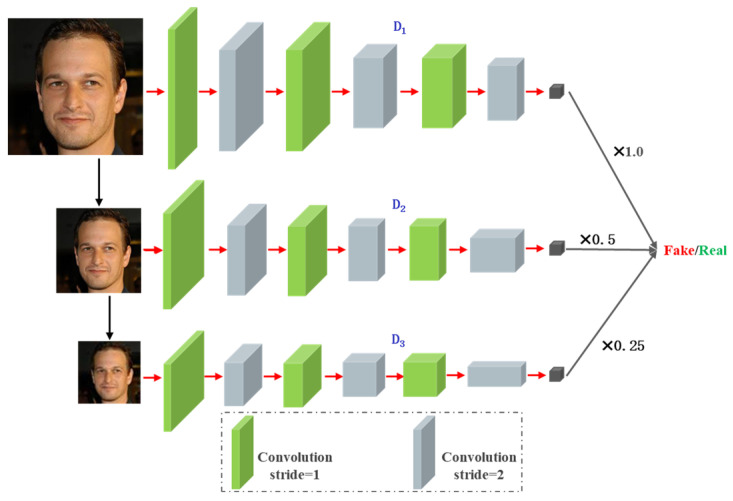
Architectural of the discriminator.

The multi-scale discriminator comprises three sub-discriminators ($D_{1},\;D_{2}\;and\;D_{3}$) structured hierarchically to process face images at resolutions of  256×256, 128×128 and 64 × 64, respectively, as shown in Figure 4.

Each sub-discriminator adopts a similar infrastructure, and they all use three downsamplings with a stride of 2. After progressive downsampling, the input images are fed into discriminators $D_{1},\;D_{2}\;and\;D_{3}$ for multi-scale analysis. The outputs of these multi-scale discriminators are combined through a weighted summation with weights $\left[1.0,\;0.5\;and\;0.25\right]$, enabling comprehensive supervision of generator training. This design forces the generator to approximate the real data distribution across both global structures (emphasized by $D_{1}$) and local textures (emphasized by $D_{3}$).

### 3.2. Loss Functions

The discriminator comprises three sub-discriminators ${\left\{D_{k}\right\}}_{k=1}^{3}$, where each sub-discriminator corresponds to an input face image downscaled by a factor of $\frac{1}{2^{k-1}}$ relative to the original resolution. The generator’s multi-scale adversarial loss aims to deceive all discriminators into classifying their inputs as “real,” whereas the discriminator’s multi-scale adversarial loss strives to distinguish between the real and generated images. Consequently, the adversarial losses for both the generator and discriminators are formally defined as follows:

(11) $$L_{adv}^{G}=\sum_{k=1}^{3}\gamma_{k}\cdot E_{z\sim P_{z}(z)}\left|{\left(D_{k}\left(G\left(z_{lr}\right)\right)-1\right)}^{2}\right|$$

(12) $$L_{adv}^{D}=\sum_{k=1}^{3}\gamma_{k}\cdot (E_{x\sim P_{data}}\left|{\left(D_{k}\left(x_{hr}\right)-1\right)}^{2}\right|+E_{z\sim P_{z}(z)}\left|{D_{k}\left(G\left(z_{lr}\right)\right)}^{2}\right|)$$

where $z_{lr}$ represents the LR input image, $x_{hr}$ denotes the original HR image, and $\gamma_{k}$ is the weight coefficient for the k-th sub-discriminator. The weight coefficients for different scales $\left\{\gamma_{k}\right\}$ are defined according to the following rules:

(13) $$\gamma_{k}=\frac{1}{2^{k-1}},k=1,2,3$$

Consequently, the weight coefficients are assigned as $\gamma_{1}=1.0,\;\gamma_{2}=0.5\;and\;\gamma_{3}=0.25$, where higher-resolution discriminators receive greater weights, while lower-resolution ones exhibit progressively decreasing weights.

In practical training, the generator is augmented with a perceptual loss function and an L1 loss function to enable joint optimization:

(14) $$L^{G}=\alpha \cdot L_{adv}^{G}+\beta \cdot L_{per}^{G}+\gamma \cdot L_{L1}^{G}$$

In the above equation, α, β and γ are designated as the weighting coefficients for the multi-scale adversarial loss, perceptual loss, and L1 loss function, respectively.

The total loss of the discriminator is defined as:

(15) $$L^{D}=L_{adv}^{D}$$

## 4. Experiments and Results

### 4.1. Datasets and Experimental Settings

The CelebA-HQ dataset is a publicly available dataset for computer vision research, comprising 30,000 face images with varying resolutions. The dataset includes images at five distinct scales: 64×64, 128×128, 256×256, 512×512 and 1024×1024, catering to diverse application requirements. For this study, we curated a subset of 15,000 images with resolutions of 64×64 and 256×256, with 1000 images designated as the test set.

The model training was conducted on a hardware server equipped with 8 Nvidia 3090 Ti GPUs (Santa Clara, CA, USA), running Ubuntu 18.04 and Py-Torch 2.1.0. All other hyperparameters are listed in Table 1.

During the initial phase of generator training, a larger learning rate (Lr) is employed to rapidly converge toward the local minimum of the loss function. Given that face image SR tasks require the restoration of high-frequency details, the Lr is progressively reduced during the later training stages to prevent parameter oscillations near the optimal value, thereby ensuring rapid convergence. The Lr for the generator is defined as follows:

(16) $$Lr(epoch)\left\{\begin{array}{l} 0.0002,\;if\;0\leq epoch\leq 100\\ 0.0001,\;if\;100<epoch\leq 200\\ 0.00005,\;if\;200<epoch\leq 300\\ 0.00002,\;if\;epoch>300 \end{array}\right.$$

The generator employs a loss function that integrates three components: a perceptual loss, an L1 loss, and a multi-scale adversarial loss. Among these, L1 loss emphasizes pixel-level errors; however, insufficient weight allocation to this term may fail to adequately constrain the generator’s output. Conversely, the multi-scale adversarial loss drives the generator to produce realistic facial images, but excessive weighting may induce color artifacts in localized regions. To address this, the training process dynamically adjusts loss weights through a phase-wise focus on distinct objectives (structural fidelity followed by detail refinement), thereby balancing L1 and multi-scale adversarial loss contributions for high-fidelity facial reconstruction. The mathematical formulations are defined as follows:

(17) $$\gamma \left(epoch\right)=0.4+\max\left(0,\frac{epoch-100}{10,000}\right)\times 2$$

(18) $$\alpha \left(epoch\right)=0.5-\max\left(0,\frac{epoch-100}{10,000}\right)\times 2$$

for epoch≤100, γ=0.4, and α=0.5. As epochs exceed 100, γ progressively increases while α decreases during training.

### 4.2. Comparative Experiments

To validate the SR performance on face images, our proposed model was compared against classical approaches, including Bicubic interpolation, SRCNN, EDSR, SRGAN, ESRGAN, Real-ESRGAN, and SwinIR. All baseline models were retrained from scratch on the same dataset without utilizing pretrained weights from the original models. The architectures of the compared models strictly adhered to the specifications described in the original papers, with minor adjustments to hyperparameters made to accommodate hardware constraints on our server (e.g., GPU memory limitations). Quantitative evaluation was conducted using the widely adopted PSNR, SSIM [31], and LPIPS [32]. Higher PSNR and SSIM values indicate greater similarity between super-resolved images and the original HR images. The lower the LPIPS value is, the higher the similarity between the images is.

**Figure 5:**
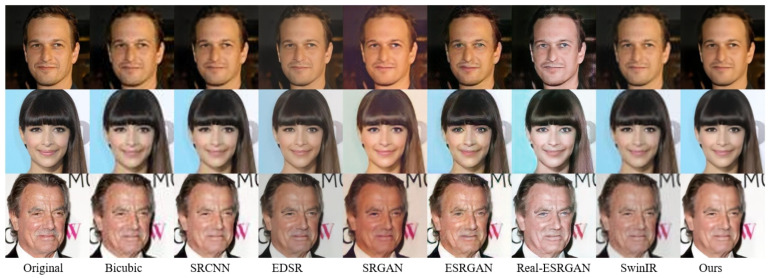
Visual comparison of super-resolved images.

Figure 5 is the comparative analysis of SR results for face images. The first column presents the original HR images, followed by super-resolved outcomes from seven benchmark models: Bicubic interpolation, SRCNN, EDSR, SRGAN, ESRGAN, Real-ESRGAN and SwinIR, respectively. The final column displays the proposed model’s results.

Bicubic interpolation, a traditional interpolation-based method, generates new pixel values using a 16-pixel neighborhood. While computationally efficient, it introduces significant distortions in high-frequency face regions, achieving the lowest performance among all methods. SRCNN, the first CNN designed for SR, employs only three convolutional layers, limiting its receptive field and restoration capability for complex face textures. EDSR incorporates a deep residual architecture, substantially improving reconstruction quality. However, its high computational complexity and tendency to produce over-smoothing artifacts in super-resolved face images remain critical limitations. SRGAN pioneered the integration of GAN into image SR, demonstrating enhanced performance relative to conventional interpolation-based approaches. However, its generated high-frequency textures exhibit overly stylized and repetitive patterns, with noticeable blurring artifacts around face regions such as the mouth. The ESRGAN model enhances the generator architecture through improved feature fusion mechanisms, yet it still suffers from structural distortions in complex face images. Real-ESRGAN employs spectral normalization and a U-Net architecture to boost discriminative capability, but the absence of perceptual loss constraints leads to blurred details in critical regions like the eyes. SwinIR introduces the Swin Transformer into the super-resolution task, improving the computational efficiency through “window-level” self-attention while maintaining good modeling ability for local and global information. Its super-resolution result is superior to the previous several methods. In contrast, our proposed improved model demonstrates optimal reconstruction performance across both global structures and fine details, aligning with human visual perception.

To conduct comparative experiments in a rigorous manner, this study performs a quantitative analysis of our proposed model against six baseline models. To test the stability of the model, we conducted three independent tests on all the models on three different test sets, that is, to obtain the means and standard deviations of PSNR, SSIM, and LPIPS. The PSNR, SSIM, and LPIPS metrics are presented in Table 2. Among these, the Bicubic model exhibits the lowest values, consistent with the qualitative comparisons shown in Figure 5. As the first GAN-based SR model, SRGAN demonstrates relatively inferior performance due to its simplistic architecture, with its metric values surpassing only baseline methods but trailing behind advanced GAN-based approaches like ESRGAN. Our improvements to the generator and discriminator, along with a phased and refined training strategy, resulted in the highest PSNR and SSIM values, as well as the lowest LPIPS in all evaluated models. In addition, in terms of the standard deviation of PSNR, SSIM, and LPIPS, the stability of our method in the three independent tests is also the best.

**Figure 6:**
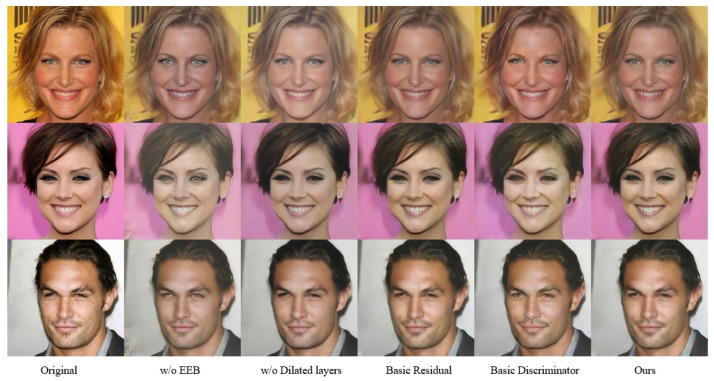
Visual comparison of ablation experiments.

This study implemented four key improvements to the model architecture: the incorporation of an Edge-guided Enhancement Block (EEB) and dilated convolution block, the redesign of standard residual blocks into Multi-scale Hybrid Attention Residual Blocks (MHARB), and the enhancement of the standard discriminator to a multi-scale discriminator. To systematically evaluate the contribution of each component in face image SR tasks, an ablation study was conducted. Qualitative comparisons of the ablation study are visualized in Figure 6, with quantitative metrics provided in Table 3.

The EEB employs a detail-adaptive enhancement strategy to effectively reduce noise while precisely restoring high-frequency textures. Experimental results prove that the absence of EEB leads to severe distortions in high-frequency details (e.g., ocular regions) of complex face images, causing visual discomfort. The multi-scale discriminator, through feature extraction across multiple scales, balances the generation quality of global structures and local details, thereby better constraining the generator to produce realistic face images. Its removal results in blurred artifacts in regions such as the eyes. The MHARB enhances the generator’s modeling capacity for critical facial features via dual-branch convolution fused with CBAM. Conversely, the removal of dilated convolution modules or the use of original residual blocks leads to a decline in PSNR and SSIM values.

**Figure 7:**
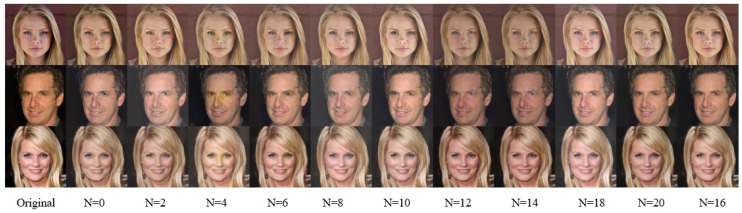
Visual comparison of residual block quantity variations.

The generator model incorporates 16 improved residual blocks following standard convolutional layers. This study assesses how the number of residual blocks affects SR performance through comparative experiments. The results in Figure 7 and Table 4 reveal that incrementally increasing residual blocks gradually enhances global color fidelity and texture sharpness in super-resolved face images. However, when N exceeds 16, the quantitative metrics decline instead of improving. Furthermore, excessive residual blocks lead to unacceptably high computational overhead. Therefore, N=16 is selected as the optimal configuration.

**Figure 8:**
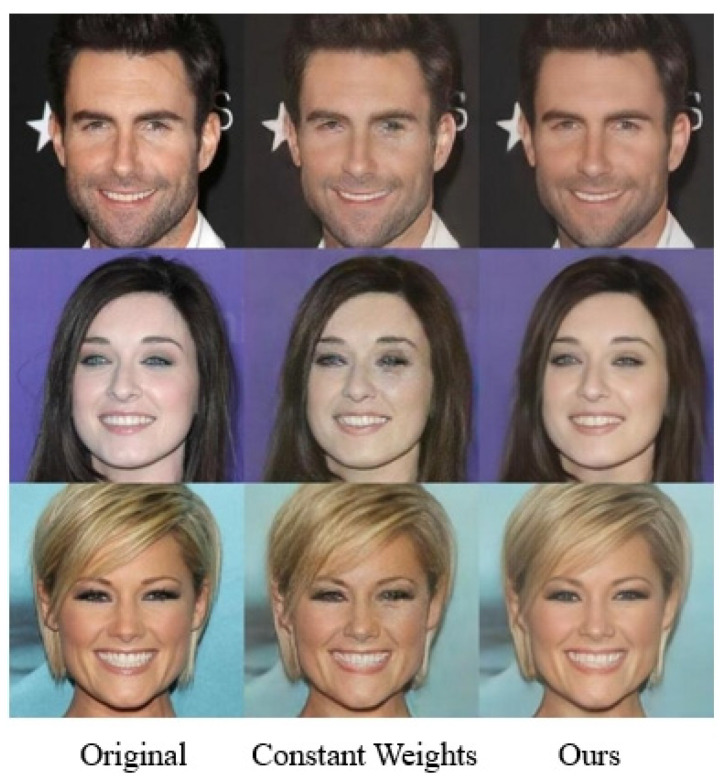
Visual comparison of dynamic weight adjustment in loss functions.

During model training, the weights of the multi-scale adversarial loss function and L1 loss function were dynamically adjusted. By progressively reducing the multi-scale adversarial loss’s weight and increasing the L1 loss’s weight, the reconstruction of high-frequency components in face images was enhanced. In Figure 8, the eyes in the second column of facial images exhibit noticeable scar-like artifacts, showing significant discrepancies compared to the original HR counterparts. Table 5 illustrates the performance improvements in PSNR, SSIM, and LPIPS. The results indicate an enhancement of 1.56 dB for PSNR, 0.0398 for SSIM, and 2.1 for LPIPS.

**Figure 9:**
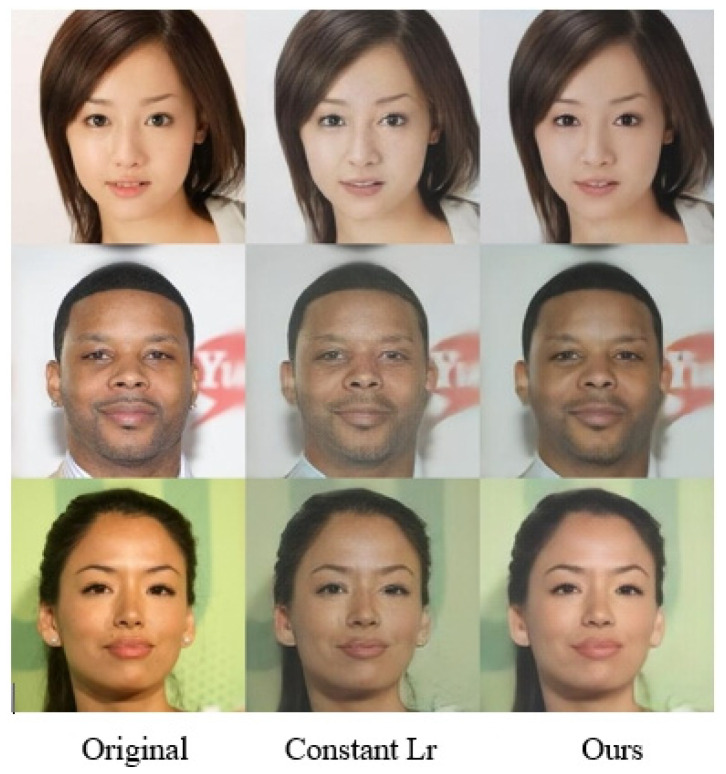
Visual comparison of dynamic adjustment in Lr.

Furthermore, during model training, a progressive reduction strategy was applied to the generator’s Lr, achieving superior SR results. Figure 9 demonstrates that the decreasing Lr strategy better preserves texture details in reconstructed images. Table 6 illustrates the performance improvements. The PSNR shows an enhancement of 2.67 dB; LPIPS is decreased by 0.49, while the SSIM rises by 0.0306, confirming the effectiveness of the proposed Lr adaptation.

To further demonstrate the generalization ability of the proposed method on different image distributions, we conducted a cross-dataset evaluation experiment on the DIV2K dataset. DIV2K is a widely-used benchmark dataset in image super-resolution tasks, featuring high-resolution images with rich texture details and complex degradation patterns. The details of the experiment retain the same hyperparameters and training settings as described in Section 4.1. The evaluation metrics also follow the same evaluation protocol (PSNR, SSIM, and LPIPS) as CelebA-HQ.

**Figure 10:**
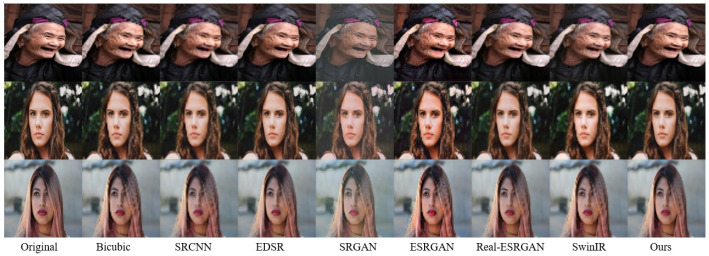
Visual comparison on DIV2K dataset.

As shown in Figure 10 and Table 7, the proposed model achieved competitive performance on DIV2K compared to other methods, demonstrating its robustness to diverse image characteristics.

## 5. Conclusions

This study proposes a novel GAN model incorporating multi-scale attention mechanisms and the Edge-guided Enhancement Block to address the insufficient restoration of high-frequency details and poor training stability in face image super-resolution tasks. Through systematic architectural improvements and a phase-wise training strategy, our model significantly enhances the quality and perceptual appeal of super-resolved images, outperforming other classical SR frameworks in experimental evaluations.

In future work, we will focus on further optimizing the model architecture through the systematic exploration of lightweight designs to reduce computational overhead. Additionally, training of the model with augmented and diverse datasets will be conducted to enhance generalization capabilities and robustness against variations in real-world scenarios.

## Figures and Tables

**Table 1:** Hyperparameters of the training.

Hyperparameters	Value	Hyperparameters	Value
Optimizer	Adam	Total epochs	1000
Initial Lr	0.0002	Batch size	190
Number of CPU threads	10	Initial α	0.5
β	0.1	Initial γ	0.4

**Table 2:** Quantitative comparison of super-resolved images.

Model	PSNR (dB)	SSIM	LPIPS
Bicubic	18.51 ± 0.42	0.5539 ± 0.151	32.84 ± 0.31
SRCNN	20.22 ± 0.22	0.6882 ± 0.045	30.13 ± 0.13
EDSR	21.31 ± 0.12	0.7231 ± 0.008	24.56 ± 0.11
SRGAN	20.79 ± 0.24	0.6548 ± 0.008	32.65 ± 0.21
ESRGAN	22.46 ± 0.25	0.7204 ± 0.025	28.58 ± 0.43
Real-ESRGAN	22.91 ± 0.13	0.7061 ± 0.007	28.51 ± 0.10
SwinIR	23.28 ± 0.14	0.7340 ± 0.006	30.48 ± 0.10
Ours	23.35 ± 0.15	0.7424 ± 0.005	24.86 ± 0.09

**Table 3:** Quantitative comparison of ablation experiments.

	PSNR (dB)	SSIM	LPIPS
w/o EEB	21.31 ± 0.24	0.7198 ± 0.004	25.34 ± 0.13
w/o Dilated Layers	22.03 ± 0.16	0.7231 ± 0.007	24.93 ± 0.08
Basic Residual	21.84 ± 0.36	0.7169 ± 0.025	26.89 ± 0.17
Basic Discriminator	21.65 ± 0.15	0.7248 ± 0.011	25.78 ± 0.09
Ours	23.35 ± 0.15	0.7424 ± 0.005	24.86 ± 0.09

**Table 4:** Quantitative comparison of residual block quantity variations.

	PSNR(dB)	SSIM	LPIPS
N = 0	19.98 ± 0.86	0.6741 ± 0.175	31.32 ± 0.51
N = 2	19.78 ± 0.75	0.6767 ± 0.150	30.33 ± 0.41
N = 4	20.28 ± 0.52	0.6849 ± 0.102	30.05 ± 0.42
N = 6	20.98 ± 0.30	0.6899 ± 0.052	27.81 ± 0.14
N = 8	19.92 ± 0.26	0.7007 ± 0.054	27.81 ± 0.16
N = 10	20.69 ± 0.25	0.7043 ± 0.047	26.49 ± 0.14
N = 12	20.85 ± 0.08	0.6895 ± 0.024	27.81 ± 0.10
N = 14	21.79 ± 0.14	0.6936 ± 0.019	28.01 ± 0.12
N = 16 (Ours)	23.35 ± 0.15	0.7424 ± 0.005	24.86 ± 0.09
N = 18	21.2 ± 0.13	0.7203 ± 0.008	24.98 ± 0.11
N = 20	20.87 ± 0.16	0.7112 ± 0.009	25.49 ± 0.17

**Table 5:** Quantitative comparison of dynamic weight adjustment in loss functions.

	PSNR (dB)	SSIM	LPIPS
Constant Weights	21.79 ± 0.16	0.7026 ± 0.005	26.96 ± 0.21
Ours	23.35 ± 0.15	0.7424 ± 0.005	24.86 ± 0.09

**Table 6:** Quantitative comparison of dynamic adjustment in Lr.

	PSNR (dB)	SSIM	LPIPS
Constant Lr	20.68 ± 0.18	0.7118 ± 0.007	25.35 ± 0.78
Ours	23.35 ± 0.15	0.7424 ± 0.005	24.86 ± 0.09

**Table 7:** Quantitative comparison on DIV2K dataset.

Model	PSNR (dB)	SSIM	LPIPS
Bicubic	23.32 ± 0.21	0.6971 ± 0.027	35.42 ± 0.26
SRCNN	23.99 ± 0.39	0.7031 ± 0.058	38.58 ± 0.20
EDSR	24.56 ± 0.26	0.7293 ± 0.017	36.82 ± 0.18
SRGAN	19.20 ± 0.27	0.5782 ± 0.018	43.87 ± 0.24
ESRGAN	23.56 ± 0.16	0.7184 ± 0.013	30.58 ± 0.28
Real-ESRGAN	22.45 ± 0.12	0.6924 ± 0.019	33.54 ± 0.14
SwinIR	24.71 ± 0.14	0.7199 ± 0.006	36.45 ± 0.10
Ours	25.24 ± 0.09	0.7201 ± 0.005	30.48 ± 0.06

## Data Availability

The data utilized in this study are sourced from publicly available datasets, accessed at the link https://gitcode.com/Resource-Bundle-Collection/8a929/tree/main (accessed on 14 December 2023).
